# Identification of HDAC9 as a viable therapeutic target for the treatment of gastric cancer

**DOI:** 10.1038/s12276-019-0301-8

**Published:** 2019-08-26

**Authors:** Kai Xiong, Hejun Zhang, Yang Du, Jie Tian, Shigang Ding

**Affiliations:** 1 Department of Gastroenterology, Peking University Third Hospital, Beijing, 100191 China; 2 CAS Key Laboratory of Molecular Imaging, The State Key Laboratory of Management and Control for Complex Systems, Institute of Automation, Chinese Academy of Sciences, Beijing, 100190 China; 3 Beijing Key Laboratory for Helicobacter Pylori Infection and Upper Gastrointestinal Diseases, Beijing, 100191 China; 4 University of Chinese Academy of Sciences, Beijing, 100080 China; 5 Beijing Advanced Innovation Center for Big Data-Based Precision Medicine, School of Medicine, Beihang University, Beijing, 100191 China; 6 Engineering Research Center of Molecular and Neuro Imaging of Ministry of Education, School of Life Science and Technology, Xidian University, Xi’an, Shaanxi 710126 China

**Keywords:** Cancer imaging, Prognostic markers

## Abstract

Histone deacetylase inhibitors (HDACis) are a new class of anticancer drugs confirmed to have good therapeutic effects against gastric cancer (GC) in preclinical experiments, but most HDACis are non-selective (pan-HDACis), with highly toxic side effects. Therefore, it is necessary to screen HDAC family members that play key roles in GC as therapeutic targets to reduce toxic side effects. In this study, we evaluated the targeting specificity of the HDACi suberoylanilide hydroxamic acid (SAHA) for GC via fluorescence molecular imaging (FMI). In vitro FMI results showed that SAHA had higher binding affinity for GC cells than for normal gastric cells. In vivo FMI of gastric tumor-bearing mice confirmed that SAHA can be enriched in GC tissues. However, there was also a high-concentration distribution in normal organs such as the stomach and lungs, suggesting potential side effects. In addition, we found that among the HDAC family members, HDAC9 was the most significantly upregulated in GC cells, and we verified this upregulation in GC tissues. Further experiments confirmed that knockdown of HDAC9 inhibits cell growth, reduces colony formation, and induces apoptosis and cell cycle arrest. These results suggest that HDAC9 has an oncogenic role in GC. Moreover, HDAC9 siRNA suppressed GC tumor growth and enhanced the antitumor efficacy of cisplatin in GC treatment by inhibiting the proliferation and inducing the apoptosis of GC cells in vitro and in vivo. Our findings suggest that the development of HDAC9-selective HDACis is a potential approach to improve the efficacy of chemotherapy and reduce systemic toxicity.

## Stomach cancer: identifying the right target

Inhibiting histone deacetylase 9 (HDAC9), a protein that regulates gene expression, reduces stomach cancer cell growth. The efficacy of current treatments for stomach cancer is limited. Although HDACs have emerged as promising therapeutic targets, non-selective HDAC inhibitors can cause severe side effects. Shigang Ding at Peking University Third Hospital in Beijing, China, and colleagues found that human stomach cancer cells have significantly higher levels of HDAC9 than other members of the HDAC family and that high HDAC9 levels are associated with reduced patient survival. Interfering with the production of HDAC9 protein improved the efficacy of the chemotherapeutic drug cisplatin in mice with stomach cancer. The authors suggest that selective HDAC9 inhibitors could help to improve the survival of patients with this type of cancer.

## Introduction

Over 723,000 patients die from gastric cancer (GC) each year worldwide, making it the third leading cause of cancer-related death^1^. Approximately 60% of patients are in an advanced stage at diagnosis, with poor prognosis. The 5-year survival rates for regional GC and GC with distant metastasis are only 30.6% and 5.3%, respectively, according to the 2018 SEER data from the National Cancer Institute (NCI). Chemotherapy can relieve symptoms and improve the survival and quality of life of patients with advanced or metastatic GC. Several agents have shown anti-GC activity individually and in combination, including fluorouracil, cisplatin, irinotecan, paclitaxel, and docetaxel^2–5^. At present, the first-line treatment for patients with advanced GC is mainly based on a 5-fluorouracil and cisplatin chemotherapy regimen. However, the efficacy of this regimen is limited, and only 30–40% of patients respond to this treatment^6^. It is therefore necessary to further explore the pathogenesis of GC and identify novel therapeutic targets and agents.

Histone deacetylases (HDACs) are a class of histone modification enzymes that regulate the expression of genes via the removal of acetyl groups from lysine residues located on the amino-terminal tails of histone proteins. Owing to their essential role in gene expression regulation, HDACs are broadly involved in cell cycle regulation, differentiation, growth, and other cellular events^7^. Studies have shown that the function or expression of HDACs is often perturbed in various cancers^8^; for example, high expression of histone deacetylase 5 (HDAC5) in breast cancer has been associated with inferior prognosis^9^. Another study showed that HDAC1 expression was upregulated in gallbladder cancer and associated with lymph node metastasis and poor overall survival^10^. In addition, HDAC10 is highly expressed in lung cancer tissues and can promote lung cancer proliferation^11^. The drastic increase in published work on HDACs indicates that these proteins are in the spotlight of cancer research and have emerged as promising cancer biomarkers and therapeutic targets^12^. However, few studies have explored the expression and therapeutic effects of HDACs in GC.

HDAC inhibitors are a new class of antitumor drugs. Treatment with HDAC inhibitors (HDACis) can restore the balance of histone acetylation and deacetylation in tumor cells. Vorinostat, known as SAHA, is a small molecule compound that inhibits HDACs by binding to their active sites^13^. The use of SAHA against refractory cutaneous T-cell lymphoma is approved by the US Food and Drug Administration (FDA), and SAHA is experiencing limited use in some solid tumors^14,15^. Recent studies have shown that SAHA can kill GC cells both in vitro and in vivo, indicating its potential in GC therapy^16^. Molecular mechanisms of the antitumor activity of SAHA, including the promotion of cycle arrest and cell apoptosis, have been described. However, little is known about the targeting and specificity of SAHA in GC that may cause its potential toxic side effects.

The aims of this study were to evaluate the affinity and specificity of the small molecule HDACi SAHA in GC and to identify potential therapeutic targets for GC treatment among the HDAC family. For this purpose, we developed fluorescently labeled SAHA probes and examined the specificity and affinity of SAHA in vitro in GC cells and in vivo in mice with gastric tumor xenografts. We then examined the expression of HDACs in GC and analyzed the molecular functions of the major HDAC with aberrant expression. Moreover, we evaluated the efficacy of the HDAC as a therapeutic target in GC.

## Materials and methods

### Cell culture

GC cell lines (BGC-823, SGC-7901, and MKN-45) and a human gastric epithelial cell line (GES-1) were purchased from the Cell Bank of the Chinese Academy of Sciences and Genechem Co., Ltd. (Shanghai, China), respectively. All cell lines were cultured in RPMI 1640 medium supplemented with 10% fetal bovine serum (Gibco, Life Technologies, USA) and 1% penicillin–streptomycin antibiotics and incubated at 37 °C in a humidified atmosphere of 5% CO_2_.

### Flow cytometric assay for SAHA affinity

Cells were cultured to 90% confluence and were then incubated with varying concentrations of fluorescein isothiocyanate-suberoylanilide hydroxamic acid (FITC-SAHA) or free FITC for 4 h. Cells were then washed twice with phosphate-buffered saline (PBS) and suspended in 200 μl of PBS. Fluorescence was analyzed using a C6 Flow Cytometer System (BD Biosciences, USA). All experiments were repeated three times.

### Mouse GC xenograft model

BGC-823 or SGC-7901 cells (5 × 10^6^ cells/100 µl PBS) were injected subcutaneously into the right upper limb of 4–5-week-old male BALB/c nude mice. When the tumors grew to 5–10 mm in diameter, the tumor xenografts were exposed for in vivo FMI imaging. After tumor imaging, the animals were killed. The tumor tissues were then removed and fixed in formalin for histopathological examination. All experimental protocols were approved by the Institutional Animal Care and Use Committee of Peking University (Permit No: 2011–0039). All experiments were performed according to the approved guidelines.

### Whole-body fluorescence molecular imaging (FMI)

In vivo targeted imaging of IRDye800CW-SAHA in gastric tumor-bearing mice was performed using an IVIS spectrum imaging system (PerkinElmer, USA). The mice (*n* = 5/group) were anesthetized, and the IRDye800CW-SAHA probe was injected via the tail vein. The blocking group was established as the control, and these mice were coinjected with IRDye800CW-SAHA and a 100-fold dose of unlabeled SAHA. Then, FMI was carried out at different time points post injection. After whole-body fluorescence imaging, the tumors and major organs were dissected to assess the FMI signal distribution ex vivo.

### Quantitative real-time PCR (qPCR)

Our qPCR method was based on previous experiments^17^. The mRNA expression of genes was normalized to that of the internal GAPDH control, and the fold changes were obtained by the relative quantification (2^−ΔΔCt^) method. The primers used for gene detection are listed in Supplementary Table 1.

### Western blotting

Western blotting was performed according to previously published procedures^17^. Primary antibodies against human HDAC9 (rabbit anti-human; Abcam, USA) and GAPDH (mouse anti-human; Beyotime, China) were used at a dilution of 1:1,000.

### Tissue microarray (TMA) immunohistochemistry (IHC) assays

A tissue microarray was obtained from Shanghai Outdo Biotech Co., Ltd. (China) with 15 paired gastric adenocarcinoma and paracancer tissues. Antigen retrieval was performed in citrate buffer by microwaving, and endogenous peroxidase activity was blocked with 3% hydrogen peroxide. Slides were then incubated with a primary rabbit anti-human HDAC9 antibody (1:500, Abcam, USA) at 37 °C for 2 h and further treated with an anti-rabbit immunohistochemistry kit (ZSGB-BIO, China). Finally, sections were stained with DAB solution and counterstained with hematoxylin. HDAC9 expression was assessed by the IHC score. The proportion score was based on the ratio of positively stained cells to gastric gland cells (0, < 5%; 1, 5–25%; 2, 26%–50%; 3, 51%–75%; 4, > 75%). The intensity score was defined as 0 for no IHC signal intensity, 1 for weak, 2 for moderate, and 3 for strong. The final IHC score for HDAC9 was calculated by multiplying the proportion and intensity scores and ranged from 0 to 12. Finally, the GC tissues were divided into three groups according to the HDAC9 IHC score (low, 0–4; intermediate, 5–7; high, 8–12). The scores were assessed independently by two pathologists blinded to the patients’ clinicopathological data.

### HDAC9 silencing in GC cells

We knocked down HDAC9 in GC cells using a validated HDAC9-specific siRNA (siHDAC9)^18^. A nontargeting siRNA sequence (siNC) and mock infection with only the transfection reagent (Mock) were used as controls. Transfection of GC cells with siRNAs was carried out using siRNA duplexes at a final concentration of 50 nmol/L and Lipofectamine 2000 reagent. The siRNA sequences are listed in Supplementary Table 1.

### Colony formation and cell growth curve assays

Cells were seeded in six-well plates at 800 cells per well in complete RPMI 1640 24 h after transfection. The medium was replaced every 4–5 days. After 10–12 days of incubation, cells were stained with 0.1% crystal violet, and the number of colonies consisting of > 50 cells was counted.

Cell growth curve was performed using the xCELLigence system (Roche Applied Science, USA). Cells were plated at 10,000–30,000 cells per well in complete RPMI 1640. A unitless parameter termed the cell index was used to represent the number of attached cells. The cell index was recorded once every minute for the first 2 h and once every hour for the following 3–5 days.

### Apoptosis and cell cycle analysis

Cells were plated in 12-well plates and transfected with siRNA. Cell apoptosis was determined 60 h after transfection using an Annexin V-PE/7-aminoactinomycin D staining kit (BioLegend, USA). Cell cycle distribution was assessed by flow cytometry after staining with propidium iodide (KeyGEN, Nanjing, China).

### Cell Counting Kit-8 (CCK-8) assay

BGC-823 and SGC-7901 cells (6 × 10^3^/well) were seeded into 96-well plates. On the following day, the medium was replaced, and the cells were transfected with HDAC9 siRNA or control siRNA for 24 h. The transfected cells were then treated with different concentrations of cisplatin. After further incubation for 24–72 h, CCK-8 reagent was added to each well and incubated for 2 h. Then, the optical density (OD) value at a wavelength of 450 nm was measured. Cells not transfected with siRNA were used as the control. The cell viability index was calculated according to the following formula: experimental OD value/control OD value × 100%. The experiments were repeated three times.

### In vivo antitumor studies

The tumor model was established as described above. Tumor volumes were measured according to the following formula: 1/2 × length × width^2^. When the tumors reached ~85 mm^3^, the mice were randomly assigned to six groups (*n* = 5) and injected with 1 nmol siNC, 1 nmol siHDAC9, 1 nmol siNC combined with 2 mg/kg or 4 mg/kg cisplatin, or 1 nmol siHDAC9 combined with 2 mg/kg or 4 mg/kg cisplatin. Cholesterol-conjugated siRNA in 0.1 ml of saline was injected into the tumor masses twice a week. Mice in the cisplatin treatment group were injected intraperitoneally with cisplatin in 0.2 ml of saline the next day after the siRNA injection. The mice were monitored until the tumors in the siNC control group mice reached 1500 mm^3^. Then, the tumors were removed and fixed with 10% buffered formalin for further analysis. All experiments were performed according to the institutional guidelines.

### Immunohistochemistry

The IHC procedures were the same as described above in the TMA IHC experiment. Primary antibodies against human HDAC9 (rabbit anti-human; Abcam, USA) and Ki-67 (rabbit anti-human; Affinity Biosciences, USA) were used at dilutions of 1:500 and 1:200, respectively.

### TUNEL assay

Tumor sections were labeled with an In Situ Cell Death Detection Kit (Roche, Shanghai, China) following the manufacturer’s instructions.

### Statistical analysis

Statistical analysis was performed using GraphPad Prism, version 7.0 (GraphPad Software, Inc., USA). The results from three independent experiments are presented as the means ± standard deviations. Differences between the experimental and control groups were analyzed using the two-tailed Student’s *t* test. Statistical significance was inferred for *P* < 0.05.

## Results

### The antiproliferative effect of SAHA on GC cells

**Fig. 1:**
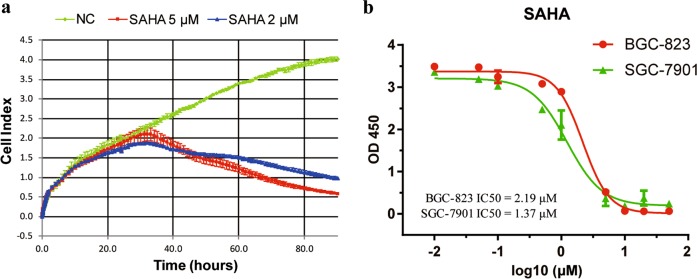
The effect of SAHA on the proliferation of gastric cancer cells. **a** A real-time cell proliferation assay using the xCELLigence system showed that SAHA treatment induced the death of BGC-823 cells in both a concentration- and time-dependent manner. **b** Determination of the IC50 of SAHA in BGC-823 and SGC-7901 GC cells treated with SAHA for 72 h

Previous studies showed that HDACs were abnormally expressed in GC^19–22^ and that pan-HDACis had a therapeutic effect in GC^16,23^. Therefore, HDACs may be potential therapeutic targets for GC. Our data showed that SAHA, a pan-HDACi, effectively inhibited GC cell growth in both a concentration- and time-dependent manner (Fig. 1a). The proliferation of BGC-823 and SGC-7901 GC cells was inhibited by SAHA, with IC50 values of 2.19 μm and 1.37 μm, respectively (Fig. 1b).

### Binding affinity of SAHA for GC cells

**Fig. 2:**
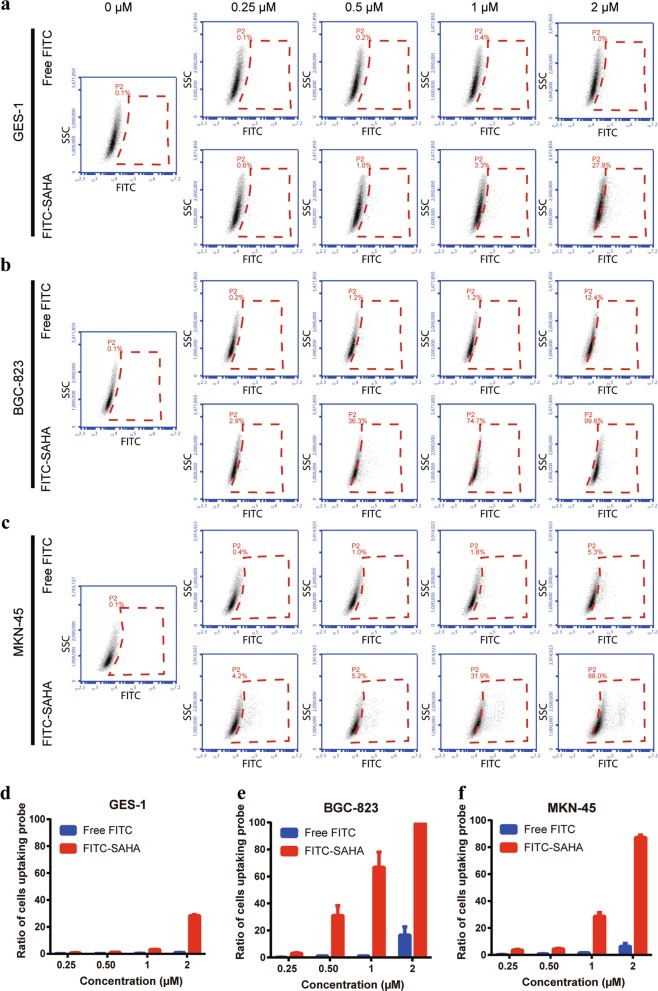
Binding capacity and affinity of SAHA for GC cells. **a–c** The GES-1, BGC-823, and MKN-45 cell lines were incubated with various concentrations of FITC-SAHA for 4 h. Fluorescence was analyzed by flow cytometry. GES-1, BGC-823, and MKN-45 cells treated with various concentrations of free FITC were used as controls. **d–f** The proportion of positive cells labeled by FITC-SAHA or free FITC was calculated. All experiments were repeated three times

The binding capacity and affinity of FITC-labeled SAHA for GC cells was assessed by flow cytometry. The flow cytometry data showed that the percentage and fluorescence intensity of positive cells in the P2 gate steadily increased with increasing concentrations of FITC-SAHA, wheresas there was a negligible change in the percentage with increasing concentrations of free FITC (Fig. 2a–c). The mean percentages of positive cells incubated with 1 μm FITC-SAHA or free FITC were 66.9% and 1.2%, respectively, in BGC-823 cells and 28.8% and 1.7%, respectively, in MKN-45 cells (Fig. 2e, f). In addition, we observed a stronger affinity of the SAHA probe for GC cells than for normal gastric mucosal cells (GES-1). The mean percentage of positive GES-1 cells was only 3.2% when treated with 1 μm FITC-SAHA. This percentage was significantly less than that of BGC-823 and MKN-45 cells (Fig. 2d–f). Fluorescence imaging of GC cells with FITC-SAHA also showed that SAHA was mainly enriched in cell nuclei and that the fluorescence signal was distinctly brighter in GC cells than in GES-1 cells (Supplementary Fig. 1). These results demonstrated higher binding specificity and affinity of SAHA for GC cells than for normal gastric cells.

### In vivo near-infrared fluorescence imaging of IRDye800CW-SAHA in GC xenograft mouse models

**Fig. 3:**
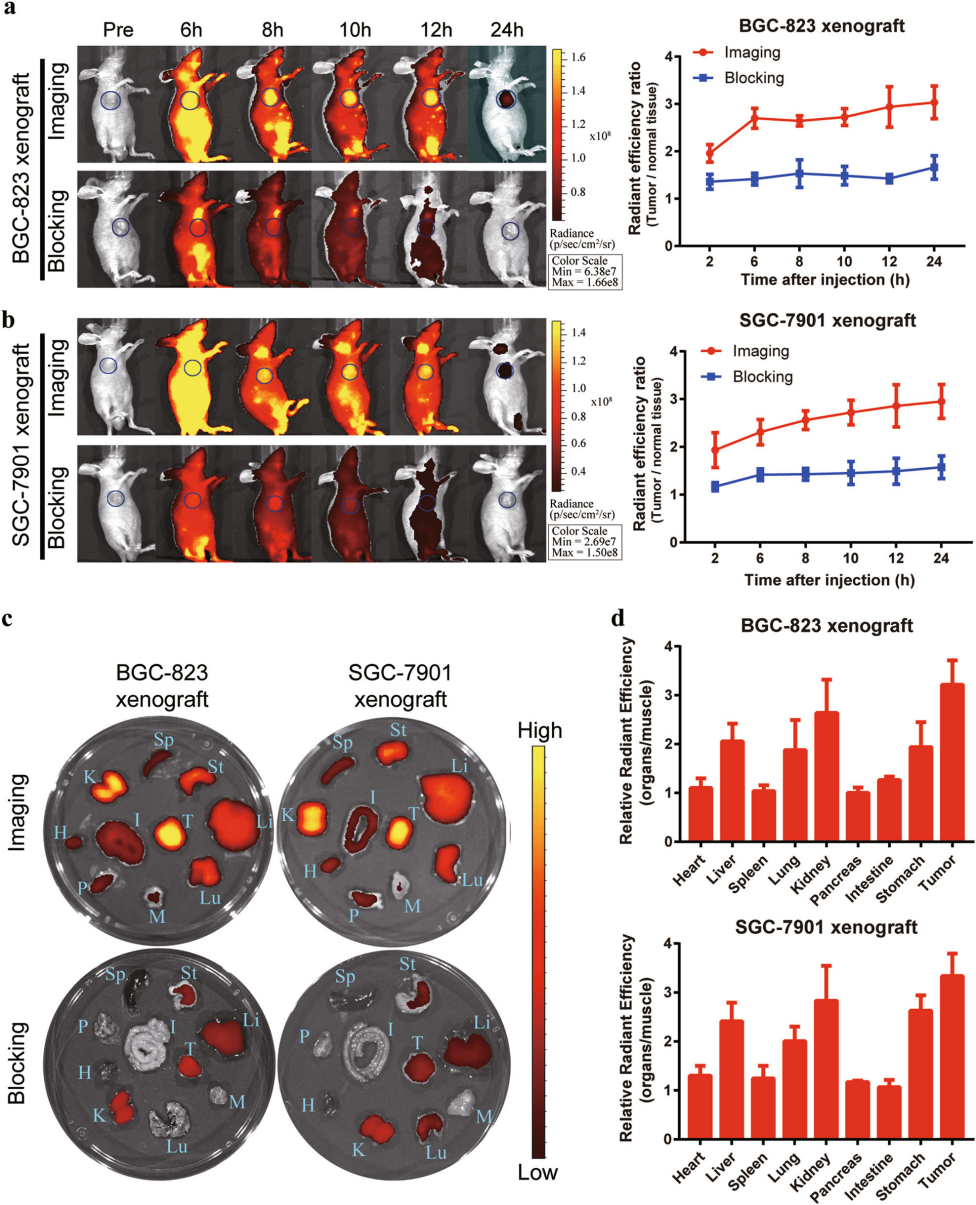
In vivo FMI of GC tumors using IRDye800CW-SAHA. **a** Specific in vivo fluorescence signals were continuously observed in mice harboring BGC-823 tumors after tail vein injection of IRDye800CW-SAHA. The signal-to-background ratios were calculated (right). **b** Similar results were observed in the SGC-7901 xenograft model. **c** Ex vivo FMI of tumors and major organs was performed after in vivo observation. A high fluorescence signal was observed in the tumors. **d** Statistical analysis of the biodistribution and fluorescence intensity of SAHA in major organs of the imaging group mice. T, tumor; Lu, lung; St, stomach; H, heart; Li, liver; Sp, spleen; K, kidney; I, intestine; P, pancreas; M, muscle

To examine the specificity of SAHA for recognizing GC cells in vivo, an IRDye800CW-labeled SAHA probe was intravenously injected into BGC-823 and SGC-7901 tumor-bearing mice, and the FMI was dynamically monitored using a small animal imaging system. Specific and increased fluorescence signals in tumors were observable 8 h after injection of the imaging probe in both the BGC-823 and SGC-7901 tumor-bearing mice, and the signals were still detectable and maintained a high signal-to-noise ratio at 24 h. In contrast, no obvious tumor accumulation was observed for the blocking group, which was coinjected with IRDye800CW-SAHA and a 100-fold dose of unlabeled SAHA (Fig. 3a, b). After in vivo imaging, the tumors and major organs were further dissected and subjected to ex vivo FMI at 48 h post injection. A high fluorescence signal was observed in the tumors from both models (Fig. 3c). Enrichment of SAHA in gastric tumors was also confirmed by confocal laser endomicroscopy (Supplementary Fig. 2). The biodistribution and fluorescence intensity of SAHA in the major organs were statistically analyzed. SAHA was mainly enriched in metabolic organs, such as the liver and kidney, but was also enriched in the lungs and stomach (Fig. 3d).

### HDAC9 is upregulated in GC cell lines and in primary GC

**Fig. 4:**
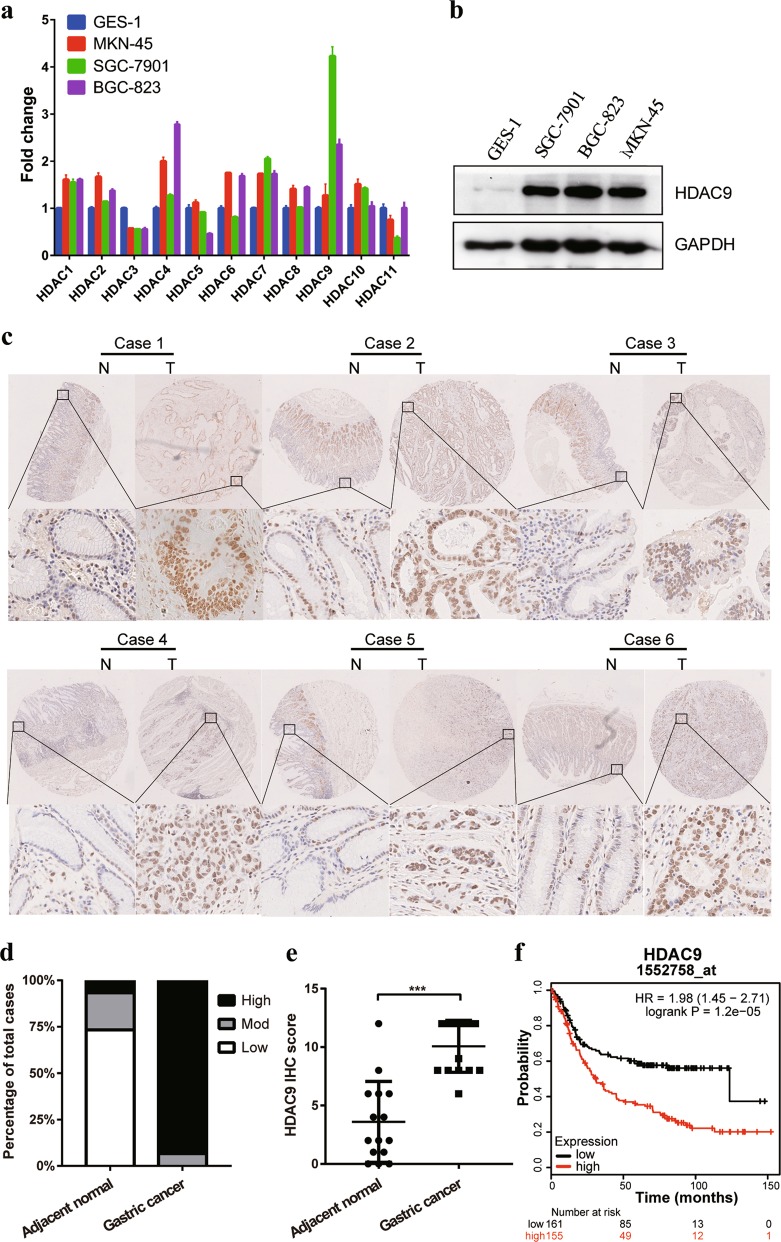
Expression of HDAC9 in GC cell lines and GC tumor tissues. **a** HDAC1–11 mRNA expression levels in the GC cell lines MKN-45, SGC-7901, BGC-823 and in the normal gastric epithelial cell line GES-1 were measured by qPCR. **b** The protein expression level of HDAC9 was confirmed by western blotting. **c** Representative HDAC9 IHC image of gastric adenocarcinoma tumor tissues (T) and paired adjacent normal tissues (N) in the tissue microarray. The area in the box was magnified × 200. **d** The proportional distribution of cases with low, moderate and high HDAC9 expression in GC tissues and the adjacent normal tissues. **e** Quantification of HDAC9 expression levels by the IHC score in GC tissues vs. adjacent normal tissues. **f** The prognostic effect of HDAC9 expression, as determined by www.kmplot.com. ***p < 0.001 versus the normal group

To determine the expression of HDACs in GC, we initially designed primers to amplify HDAC1–11 (Supplementary Table 1) and examined the mRNA expression of HDAC1–11 in human GC cell lines (SGC-7901, BGC-823, and MKN-45) and a control normal gastric epithelium cell line (GES-1) by qPCR. The results showed that the expression of most HDAC genes, except HDAC3, HDAC5, and HDAC11, was higher in GC cells than in GES-1 cells (Fig. 4a). Moreover, we found that HDAC9 showed the most significantly increased expression, with a 4.23-fold change in the SGC-7901 cell line. Hence, we focused our attention on the HDAC9 gene and further confirmed its increased protein expression in GC cells compared with that in normal GES-1 cells (Fig. 4b).

To further evaluate HDAC9 expression in clinical samples, we examined HDAC9 protein expression in GC samples by IHC in a TMA containing 15 paired GC tumor tissues and paratumor normal tissues. The expression of HDAC9 was semiquantitatively assessed by scoring the intensity of the staining and the proportion of positive cancer cells (Fig. 4c). The results showed that HDAC9 was mainly localized in cell nuclei. The HDAC9 staining intensity and proportion scores were obviously higher in GC tissues than in normal mucosal tissues. Data analysis revealed high immunoreactivity in 93.3% of tumor tissues (14/15, IHC score ≥ 8) but low expression of HDAC9 in 73.3% of adjacent normal tissues (11/15, IHC score ≤ 4), indicating an increase in HDAC9 expression in cancerous tissues (Fig. 4d, e). To further investigate the effect of aberrant HDAC9 expression on the survival of GC patients, we used the Kaplan–Meier survival plotter database (http://kmplot.com/analysis/index.php?p=service&cancer=gastric). The data showed that higher HDAC9 mRNA expression (upper quartile vs lower quartile) in GC patients was correlated with inferior overall patient survival (Fig. 4f and Supplementary Fig. 3).

### HDAC9 knockdown inhibited GC cell survival and proliferation but did not affect cell migration

**Fig. 5:**
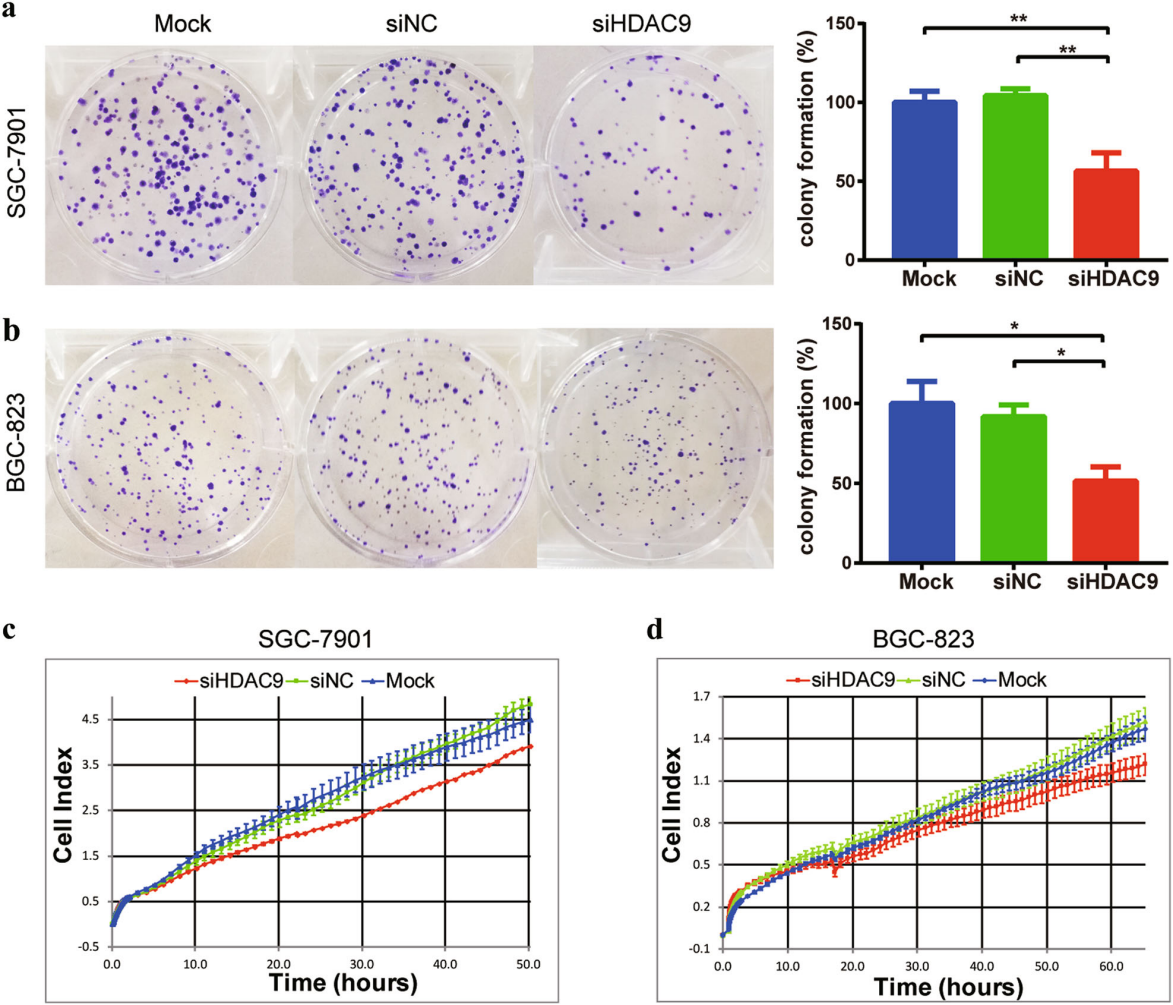
HDAC9 knockdown inhibited cell colony formation and proliferation. **a** SGC-7901 cells transiently transfected with HDAC9-specific siRNA (siHDAC9) showed a lower colony formation capacity than cells transfected with nontargeting siRNA (siNC) or mock transfection control (mock) cells. The relative colony formation numbers were calculated by comparison with the mock group. **b** Silencing of HDAC9 also suppressed colony formation in BGC-823 cells. **c**, **d** The cell index parameter used to represent the number of attached viable cells was recorded in real time. The results showed a significantly lower rate of GC cell proliferation following transfection with siHDAC9 than in cells transfected with siNC and mock transfection control cells. The error bars indicate the means ± SDs; *p < 0.05, **p < 0.01 versus the control group

To examine whether HDAC9 has an oncogenic role in GC development, we knocked down the HDAC9 gene in SGC-7901 and BGC-823 GC cells using HDAC9-specific siRNA and observed the molecular function of HDAC9 in GC cells. The silencing efficiency of HDAC9 in the transfected cells was assessed by qPCR analysis, and the data showed 83% inhibition of HDAC9 mRNA expression in BGC-823 cells after 48 h of transfection with 50 nm siHDAC9 relative to its expression in cells transfected with siNC (Supplementary Fig. 4). The colony formation assay showed notably lower colony numbers following treatment with siHDAC9 than in the siNC-treated and mock transfection groups. The magnitudes of reduction in SGC-7901 and BGC-823 cells were 44% and 49%, respectively, relative to the mock group (Fig. 5a, b). Furthermore, cell proliferation assays also showed that the proliferation of SGC-7901 cells transfected with siHDAC9 was significantly decreased (Fig. 5c). BGC-823 cells transfected with siHDAC9 also exhibited a decrease in proliferation similar to that of SGC-7901 cells (Fig. 5d). Conversely, overexpression of HDAC9 in MGC803 GC cells, with a low HDAC9 background expression level, increased cell colony formation and proliferation (Supplementary Fig. 5). In addition, we further investigated the effect of HDAC9 on cell migration. The transwell assay data showed that inhibition of HDAC9 expression did not significantly affect cell migration in the SGC-7901 cell line (Supplementary Fig. 6). Collectively, the results of the colony formation and proliferation assays suggested that HDAC9 can enhance the growth and proliferation of GC cells in vitro.

### HDAC9 silencing induced cell cycle arrest and apoptosis

**Fig. 6:**
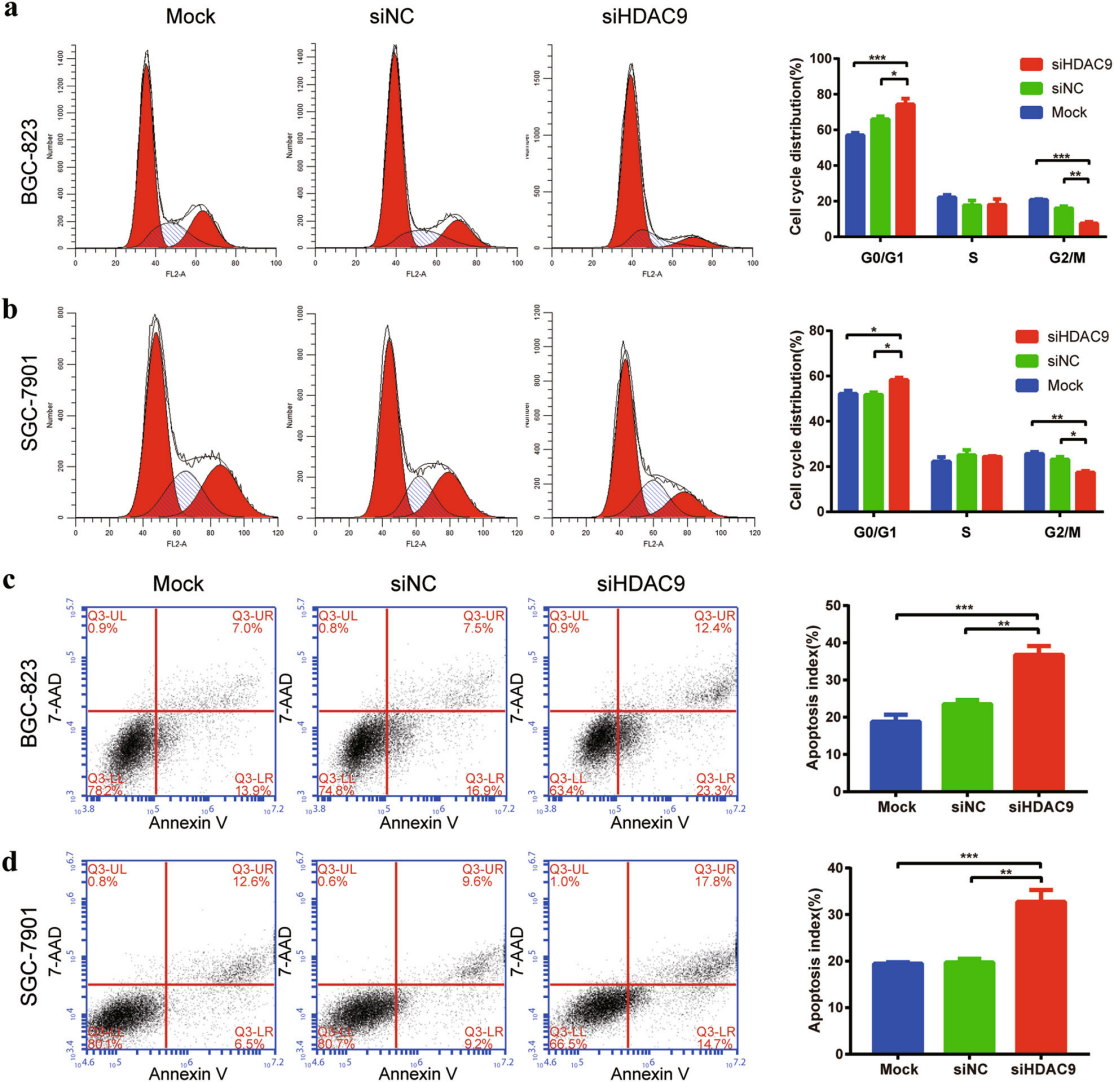
HDAC9 silencing induced cell cycle arrest and apoptosis. **a**, **b** Cell cycle analysis of PI-stained mock-, siNC-, and siHDAC9-treated cells for 48 h by flow cytometry. The histogram peaks indicate the G1/G0, S, and G2/M phases of the cell cycle. The results show that HDAC9 silencing induced G1 arrest and reduced the proportion of cells in G2/M phase. **c**, **d** HDAC9 inhibition promoted the induction of apoptosis in GC cell lines, as evidenced by the Annexin V-PE/7-AAD assay. The apoptotic index was obtained from the ratio of early to late apoptotic cells. The error bars indicate the means ± SDs; *p < 0.05, **p < 0.01, ***p < 0.001 versus the control group

To investigate the mechanism of growth inhibition by HDAC9 gene silencing, we monitored cell cycle progression by flow cytometric analysis of propidium iodide-stained DNA in GC cells. The analysis showed increased accumulation of GC cells in G0/G1 phase and a reduction in the G2/M phase population following HDAC9 silencing (Fig. 6a, b). These results indicated that HDAC9 silencing induced G0/G1 arrest and reduced the proportion of G2/M mitotic cells, leading to the inhibition of GC cell proliferation. Further investigation of the effects of HDAC9 knockdown on the apoptosis of GC cells revealed the induction of both early and late apoptosis in BGC-823 and SGC-7901 GC cells compared with the mock or siNC groups (Fig. 6c, d). Overexpression of HDAC9 in MGC803 GC cells reduced the apoptosis rate (Supplementary Fig. 5). The above results indicated that decreasing HDAC9 expression exerts an antitumor effect by inducing G0/G1 arrest and apoptosis in GC cells.

### siHDAC9 enhanced the antitumor effect of cisplatin by inhibiting the proliferation and inducing the apoptosis of GC cells

**Fig. 7:**
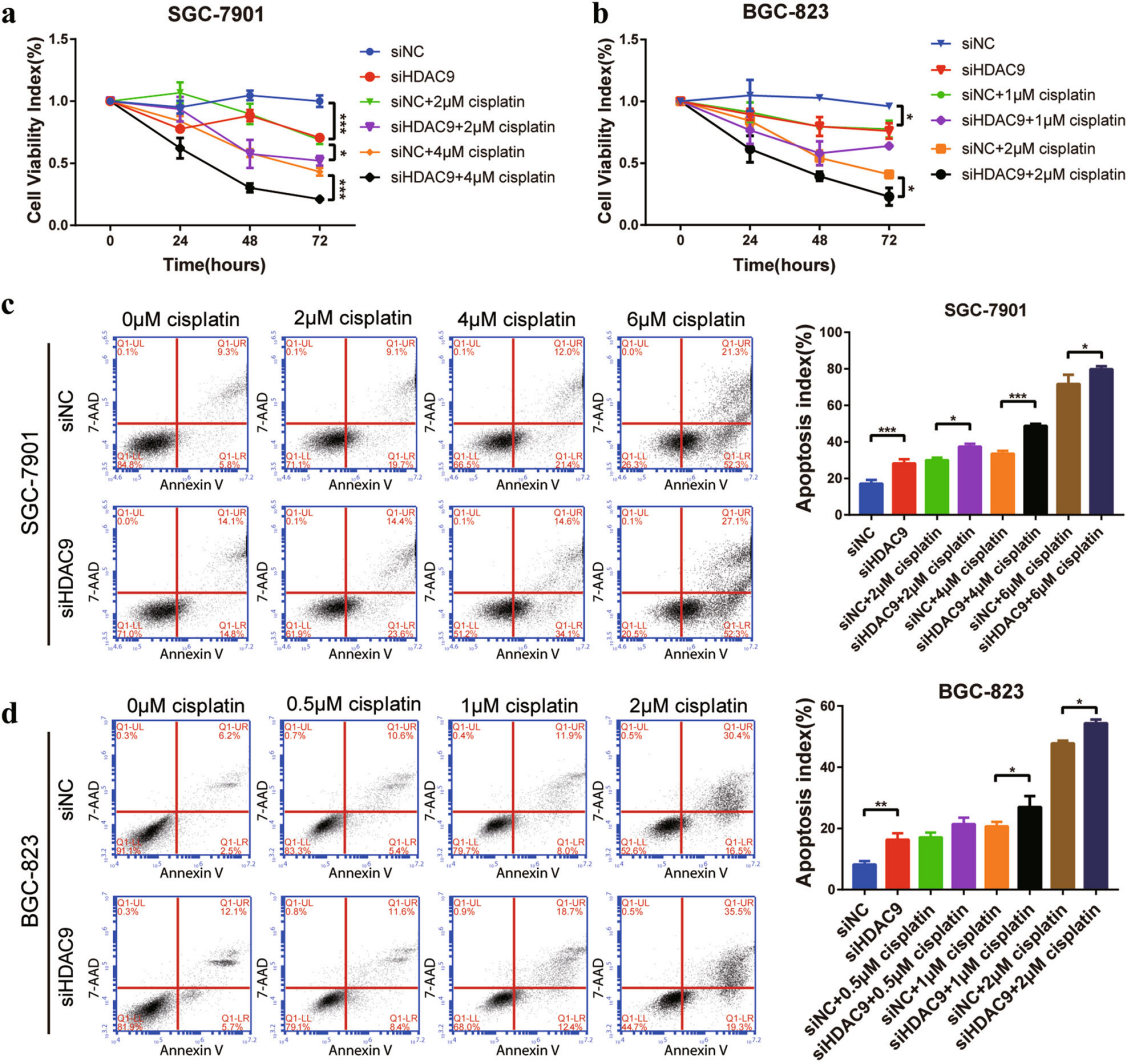
siHDAC9 enhanced the cisplatin-induced apoptosis and viability inhibition of GC cells. **a**, **b** SGC-7901 and BGC-823 cells were treated with siNC, siHDAC9, siNC + cisplatin, or siHDAC9 + cisplatin for 24–72 h. Cell viability was evaluated by a CCK-8 assay. **c**, **d** Cells were treated for 48 h. Apoptosis was detected in GC cell lines by the Annexin V-PE/7-AAD assay. The apoptotic index was obtained from the ratio of early to late apoptotic cells. The error bars indicate the means ± SDs; *p < 0.05, **p < 0.01, ***p < 0.001 versus the control group

To evaluate the therapeutic effect of siHDAC9 combined with first-line GC chemotherapeutic drugs, we treated GC cells with siHDAC9 and cisplatin and examined the effect of these treatments on GC cell growth and apoptosis. The CCK-8 assay data (Fig. 7a) showed that the combination of cisplatin and siHDAC9 had the strongest inhibitory effect on cell viability, causing reductions of 48% (siHDAC9 + 2 μm cisplatin) and 79% (siHDAC9 + 4 μm cisplatin) in the viability of SGC-7901 cells after 72 h of treatment. The single-agent treatments caused reductions of only 30% (siHDAC9), 31% (siNC + 2 μm cisplatin) and 57% (siNC + 4 μm cisplatin) in viability. The inhibitory effect of 50 nm siHDAC9 on SGC-7901 cells was almost equivalent to that of 2 μm cisplatin. The CCK-8 data for BGC-823 cells was similar to that for SGC-7901 cells and confirmed the results (Fig. 7b). In addition, compared with the single-agent treatments, combined treatment with siHDAC9 and cisplatin for 48 h significantly increased the apoptosis rate of GC cells (Fig. 7c, d). Consistent with the CCK-8 results, the apoptosis rate of SGC-7901 cells treated with 50 nm siHDAC9 was comparable to that of cells treated with 2 μm cisplatin.

### siHDAC9 enhanced the growth suppression of GC tumors in response to cisplatin in mice

**Fig. 8:**
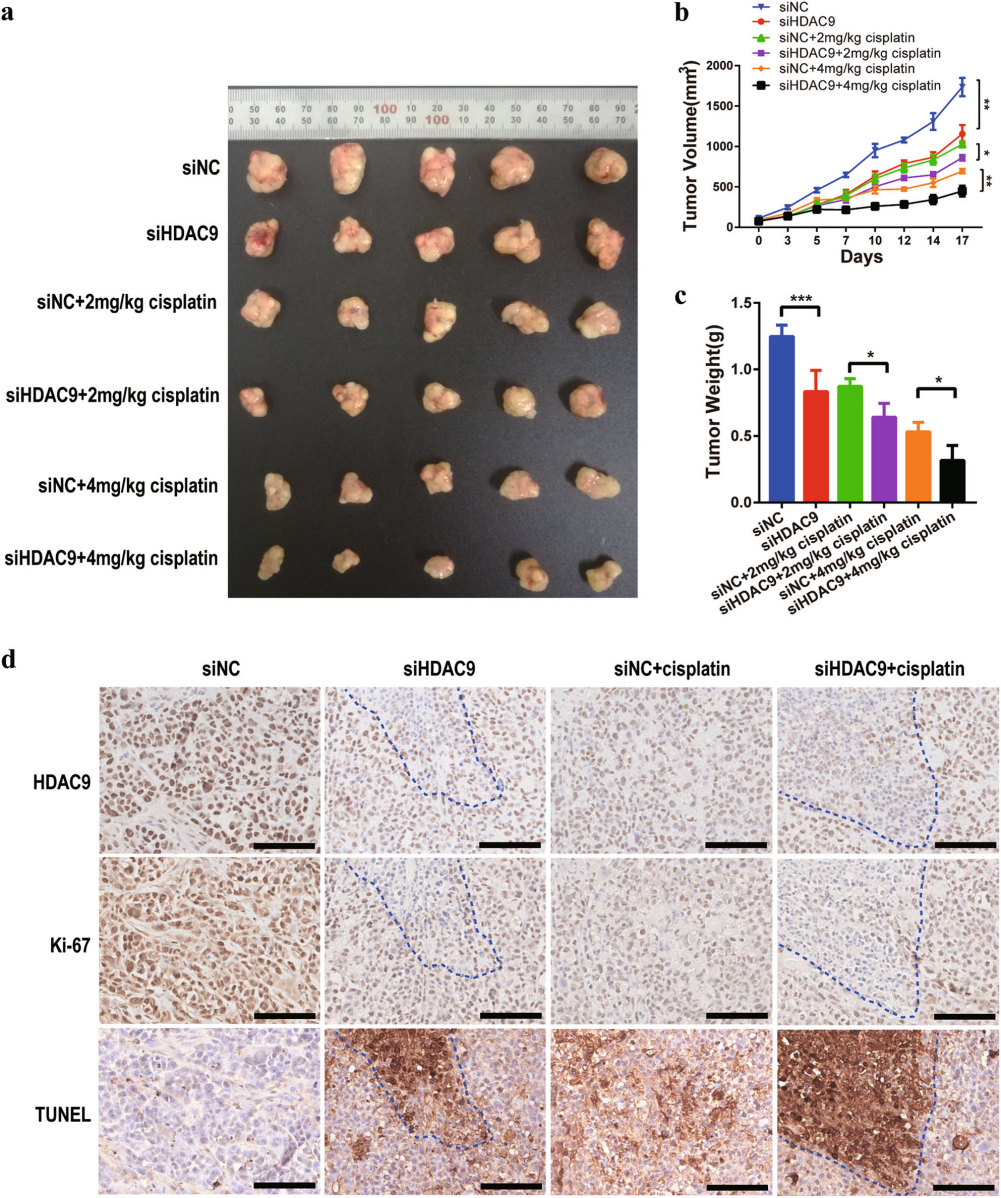
HDAC9 inhibition enhanced the effect of cisplatin on GC tumor suppression. **a** Knockdown of HDAC9 in SGC-7901 cells resulted in reduced subcutaneous tumorigenic ability and enhanced the antitumor effect of cisplatin in nude mice. **b** The tumor volumes were monitored three times weekly. The combination of siHDAC9 and cisplatin showed greater antitumor effect than each single-agent treatment. **c** The dissected xenograft tumors were weighed at the end of treatment. **d** HDAC9 expression and cell proliferation and apoptosis were assessed by HDAC9 and Ki-67 immunohistochemical staining and a TUNEL assay using serial sections. The HDAC9-negative area denoted by the dotted line indicates the location of siHDAC9 injection. Scale bars = 100 μm. The error bars indicate the means ± SDs; *p < 0.05, **p < 0.01, ***p < 0.001 versus the control group

We further evaluated the therapeutic effect of siHDAC9 combined with cisplatin on gastric tumors in vivo. The data showed that siHDAC9 combined with cisplatin showed an enhanced therapeutic effect. After 2 weeks of treatment, the tumors in the siHDAC9 and 4 mg/kg cisplatin group were significantly smaller than those in the siNC and the other single-agent treatment groups. The average tumor volumes were 447 ± 147 mm^3^, 695 ± 59 mm^3^, 1156 ± 220 mm^3^, and 1736 ± 254 mm^3^ in the siHDAC9 + 4 mg/kg cisplatin, siNC + 4 mg/kg cisplatin, siHDAC9, and siNC groups, respectively (Fig. 8a, b). In addition, the tumors in the siHDAC9 + 2 mg/kg cisplatin group were smaller than those in the siNC + 2 mg/kg cisplatin group. The removed tumors were also weighed, and the trend in the tumor weight changes was similar to that for the tumor volumes (Fig. 8c).

Tumor cell proliferation and apoptosis were further evaluated by Ki-67 immunohistochemical staining and a TUNEL assay. The groups treated with combinations of cisplatin and siHDAC9 exhibited more apoptotic cells and fewer Ki-67-positive cells than the other groups (Fig. 8d). The apoptotic cells were mainly concentrated in the region injected with siHDAC9 (HDAC9-negative area) in the siHDAC9 group, and almost all of the HDAC9-positive cells were Ki-67-positive, as evidenced by immunohistochemical staining of adjacent sections. The data showed that compared with siNC and cisplatin monotherapy, combination therapy with siHDAC9 and cisplatin resulted in the most significant reduction in proliferation and increase in apoptosis.

## Discussion

Screening for appropriate therapeutic targets and increasing the effectiveness and specificity of chemotherapeutic drugs are key to improving the quality of life and overall survival of patients with advanced and metastatic GC. The HDAC family comprises 18 HDACs grouped into four classes. These enzymes are well known for epigenetically regulating gene expression by catalyzing the removal of acetyl groups and remodeling chromatin structure. Aberrant expression of HDACs has been linked to a variety of malignancies, and high HDAC levels are frequently associated with advanced disease and poor outcomes^24,25^. HDACs have become attractive targets for chemotherapy, and a variety of HDACis have been approved by the US FDA for anticancer treatment or are in clinical trials^26^. Investigation of the specificity of HDACis and the expression patterns and functions of HDACs in tumors can help with assessing the efficacy and toxicity and improving HDACis. However, the understanding of these processes in GC is still limited.

SAHA is the first HDACi approved by the US FDA for the treatment of malignancies. Our and other researchers’ results also showed that SAHA has high GC cell-killing capability in vitro, but it failed to demonstrate efficacy in treating patients with metastatic or unresectable GC in a phase II study^27^. A key reason for this discrepancy is that the toxic side effects of vorinostat affect the choice of dosage. One explanation for this high toxicity is that not all HDAC family members are overexpressed in GC, suggesting that the inhibition of most HDACs with pan-HDAC inhibitors, including SAHA, may have unwanted toxic effects owing to the non-selective inhibition of normally expressed HDACs. It is important to evaluate the specificity of a drug before clinical trials.

To assess the targeting specificity of SAHA to GC cells, we constructed a fluorescent SAHA probe to observe the binding and biodistribution of SAHA in cells and tissues by FMI. The flow cytometry results showed that SAHA had a higher affinity for GC cells than for normal gastric cells. The in vitro FMI data showed that HDAC9 was mainly localized in cell nuclei. SAHA was also mainly concentrated in nuclei but also showed a relatively low distribution in the cytoplasm. This finding is reasonable, considering that some family members, such as HDAC6 and HDAC10, are localized in the cytoplasm^28^. Both results suggest that SAHA shows stronger targeting specificity for GC cells than for normal cells because of the higher HDAC expression in GC cells. However, these findings cannot reflect the specificity of SAHA at the tissue level. We further established GC xenograft mouse models and evaluated the binding specificity of SAHA to tumor tissues and its distribution in major organs in vivo after intravenous injection. These results showed that SAHA was significantly enriched in GC tumors but was also distributed in the liver and kidneys, presumably owing to their metabolism and excretion of SAHA. Surprisingly, there was a relatively high distribution of SAHA in the stomach. The fluorescence intensity in the stomach was comparable to that in the liver and kidneys, suggesting a potentially side effect of toxicity to normal stomach tissue because of the lack of metabolic enzymes and excretion pathways in the stomach. To date, there is still no reliable method for determining the targeting specificity of HDACis^29^. In this study, we established a method incorporating FMI to evaluate the targeting specificity of HDACi in vivo.

To identify the key gene in the HDAC family involved in the progression of GC, we quantitatively analyzed the expression of the classical HDACs in different GC cell lines and found that HDAC9 expression was the most prominently upregulated. Upregulation of the HDAC9 gene was confirmed in GC tissues by TMA analysis. HDAC9 was positively expressed in all tested GC samples; 93.3% showed high expression, and 86.7% showed HDAC9 upregulation compared with that in adjacent normal tissues. This finding indicated the notable GC specificity of HDAC9 upregulation. In addition, high HDAC9 expression was associated with shorter OS times.

Previous studies of HDAC expression patterns in GC mainly focused on class I HDACs, including the HDAC1, 2, 3, and 8 isoenzymes^19,21,30^. Weichert et al.^19^ reported IHC-assessed expression of HDAC1, HDAC2, and HDAC3 in 38.7%, 42.7%, and 53.7% of GC tissues, respectively, in a validation cohort of patients with GC. Another study showed that HDAC1 and HDAC2 were highly expressed in 54% and 85% of gastric carcinomas, respectively, but were not associated with OS^21^. One limitation of these studies was the lack of paired normal sample groups; thus, the relative change in the expression of HDACs in GC was unknown, and the specificity of HDAC expression in GC could not be satisfactorily evaluated. However, based on the available data, we can conclude that HDAC9 has higher GC specificity than HDAC1, HDAC2, and HDAC3. The above evidence indicates that HDAC9 may be a more suitable therapeutic target than other HDACs.

Several studies have confirmed that HDAC9 is involved in the development of malignant tumors. Overexpression of HDAC9 was reported in medulloblastoma, lymphoma, oral squamous cell carcinoma, retinoblastoma, and breast cancer and was shown to promote cell growth^18,31–34^. However, in contrast, another study concluded that HDAC9 was expressed at a low level in lung cancer and attenuated cell proliferation^35^. This discrepancy means that HDAC9 may play distinctly different roles in different tumors. To date, little is known about the function of HDAC9 in the development of GC. Our data showed that knockdown of HDAC9 can reduce the proliferation and clonality of GC cells. Inhibition of HDAC9 can result in the apoptosis of GC cells and induce G0/G1 phase cell cycle arrest. These results demonstrate that HDAC9 plays a tumor-promoting role in GC. Thus, inhibition of HDAC9 may be a potential strategy for the treatment of GC.

We subsequently confirmed that siHDAC9 has a certain antitumor ability. In vitro cell experiments demonstrated that the effect of 50 nm siHDAC9 on inhibiting the growth and promoting the apoptosis of SGC-7901 cells was almost equivalent to the effect of 2 μm cisplatin in GC cells. Considering the transfection efficiency and the limited ability of siHDAC9 to cross the cell membrane barrier, the antitumor effect of HDAC9 inhibition can likely be improved. In addition, both in vivo and in vitro experiments confirmed that siHDAC9 can increase the antitumor therapeutic effect of cisplatin. Immunohistochemical staining of serial sections of tumor tissues showed that Ki-67 and HDAC9 were expressed in a similar manner, suggesting that HDAC9 may be a proliferation marker. Moreover, we found that TUNEL-positive cells were mainly concentrated in the HDAC9-negative region, where siHDAC9 was injected intratumorally. It is predictable that the development of HDAC9-specific small molecule inhibitors can overcome the challenges in improving the therapeutic effect, at least enhancing the antitumor ability of the existing chemotherapeutic drug.

In summary, for the first time, we evaluated the specificity of the pan-HDACi SAHA for the treatment of GC by FMI and found that HDAC9 has an oncogenic role in GC and could be a promising therapeutic target.

## Supplementary information

Supplementary Information
