# Supplementary material for: Identification of HDAC9 as a viable therapeutic target for the treatment of gastric cancer
Source: Exp Mol Med. 2019 Aug 26;51(8):100. doi: 10.1038/s12276-019-0301-8 (PMC6802628; doi:10.1038/s12276-019-0301-8)
Supplement: Supplementary file 1 — Supplementary Information [file 12276_2019_301_MOESM1_ESM.docx]

**Supplementary Material**

Identification of HDAC9 as a viable therapeutic target for the treatment of gastric cancer

Kai Xiong^a,b,c^, Hejun Zhang^a,c^, Yang Du^b,d*^, Jie Tian^b,d,e,f*^, Shigang Ding^a,c*^

^a^ Department of Gastroenterology, Peking University Third Hospital, Beijing 100191, China

^b^ CAS Key Laboratory of Molecular Imaging, The State Key Laboratory of Management and Control for Complex Systems, Institute of Automation, Chinese Academy of Sciences, Beijing 100190, China

^c^ Beijing Key Laboratory for Helicobacter Pylori Infection and Upper Gastrointestinal Diseases, Beijing 100191, China

^d^ University of Chinese Academy of Sciences, Beijing, 100080, China

^e^ Beijing Advanced Innovation Center for Big Data-Based Precision Medicine, School of Medicine, Beihang University, Beijing, 100191, China

^f^ Engineering Research Center of Molecular and Neuro Imaging of Ministry of Education, School of Life Science and Technology, Xidian University, Xi’an, Shaanxi, 710126, China

*Corresponding Authors:

Shigang Ding: No. 49 Road Huayuan North, Haidian District, Beijing, 100191, China. E-mail: dingshigang222@163.com, Phone: +86 10 82264646, Fax: +86 10 62017700;

Jie Tian: No. 95 Road Zhongguancun East, Haidian District, Beijing, 100190, China. E-mail: jie.tian@ia.ac.cn, Phone: +86 10 82618465, Fax: +86 10 62527995;

Yang Du: No. 95 Road Zhongguancun East, Haidian District, Beijing, 100190, China. E-mail: [yang.du@ia.ac.cn](mailto:yang.du@ia.ac.cn), Phone: +86 10 62611658, Fax: +86 10 62611658.

**SUPPLEMENTARY METHODS**

*In vitro confocal microscopy imaging*

Cells were seeded in flat-bottomed 6-well plates (Costar, Corning, USA), and on the following day, incubated with 2 μM FITC-SAHA for 4 h. After washing, cells were fixed with 4% paraformaldehyde and stained with anti-HDAC9 (1:4,000 dilution) primary antibody for 1 h at room temperature in the dark, followed by anti-rabbit IgG secondary antibody conjugated with Alexa Fluor 594 (1:1000 dilution, CST, USA) in the dark for 1 h. Cells were then washed three times with PBS, and mounted with antifade mounting medium with DAPI. Images were captured using a Carl Zeiss LSM 780 confocal laser scanning microscope.

*In Vivo confocal laser endomicroscopic (CLE)Imaging*

In vivo CLE imaging was performed on the mice (n=3/group) 8 hours after FITC-SAHA and Evans blue injection using a probe-based CLE system (Cellvizio® Mauna Kea Technologies), with excitation at 488 nm and 660 nm. After mice were anesthetized, the tumors were exposed, and the confocal probe was positioned on the tumor surface and the fluorescence signal was scanned.

*Transwell migration assay*

6×10^4^ cells transfected for 24 hours were plated in serum-free DMEM in the top chamber (24-well insert; Corning, USA), while medium containing 10% FBS was used as the chemoattractant in the lower chamber. The cells were then incubated for 16 h at 37˚C. Cells on the lower surface were fixed and stained with 0.1% crystal violet. The stained cells were counted in 6 random fields under a light microscope.

**SUPPLEMENTARY TABLE**

Supplementary table 1. Sequence of primers for qRT-PCR and siRNA for HDAC9

| **Name** | **Forward sequence** **(5'to3')** | **Reverse sequence** **(5'to3')** |
| --- | --- | --- |
| **primers** |  |  |
| HDAC1 | CCAATGCTGAGGAGATGACCA | CTTGCCACAGAACCACCAGTA |
| HDAC2 | ATGGCGTACAGTCAAGGAGG | TCATGCGGATTCTATGAGGCT |
| HDAC3 | GAGAGTCAGCCCCACCAATA | GTTGTTCAGCTGGGTTGCTC |
| HDAC4 | AGCGTCCGTTGGATGTCAC | CCTTCTCGTGCCACAAGTCT |
| HDAC5 | CTCCTCTGGGTGGCTACTCT | TCACAGATGGCGGTCAAGTC |
| HDAC6 | GAGGGAGAACTCCGTGTCCTA | AATAGCCATCCATAAGACTGTGC |
| HDAC7 | TCCTGAGTACCTGGCTGCTT | CATCAGTTGCTGCGTCATGT |
| HDAC8 | CTGCCCAATGCCTGATTGAC | TGGAGGTGAAACTGAATGCGT |
| HDAC9 | GGCTCAGCAAAGAATGCACAG | CTTCTCACGGACAACAGGGT |
| HDAC10 | TGACAACGCCGGATATCACA | CCTCTCCGAACAGCCACATC |
| HDAC11 | TTCCTCCCCAACTTCCTTGTG | AACTTGATGGCGAGCGTGAT |
| GAPDH | AGAAGGCTGGGGCTCATTTG | AGGGGCCATCCACAGTCTTC |
| **siRNA** |  |  |
| siHDAC9 | GCUCAAUGCUUCGAAUUCATT | UGAAUUCGAAGCAUUGAGCTT |
| siNC | UUCUCCGAACGUGUCACGUTT | ACGUGACACGUUCGGAGAATT |

**SUPPLEMENTARY FIGURE**

**SFig.1**


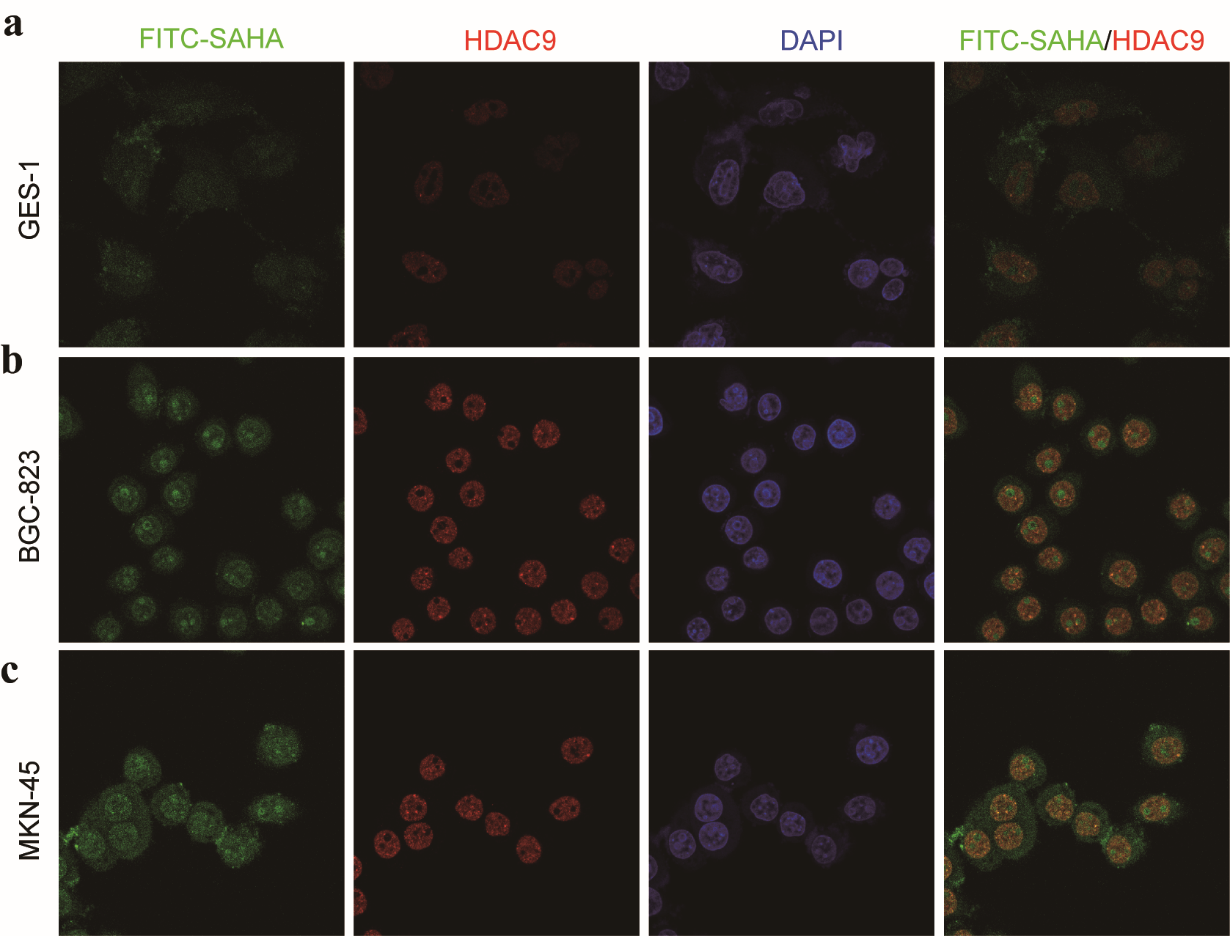


SFig.1 Fluorescence imaging of GC cells with FITC-labeled SAHA. a One normal gastric mucosa cell line, GES-1, and b, c two GC cell lines BGC-823 and MKN-45, were incubated with 2 μM FITC-SAHA for 4 h, and then observed under a confocal fluorescence microscope. The localization and intensity of HDAC9 expression in cells was also visualized by immunofluorescence.

**SFig.2**


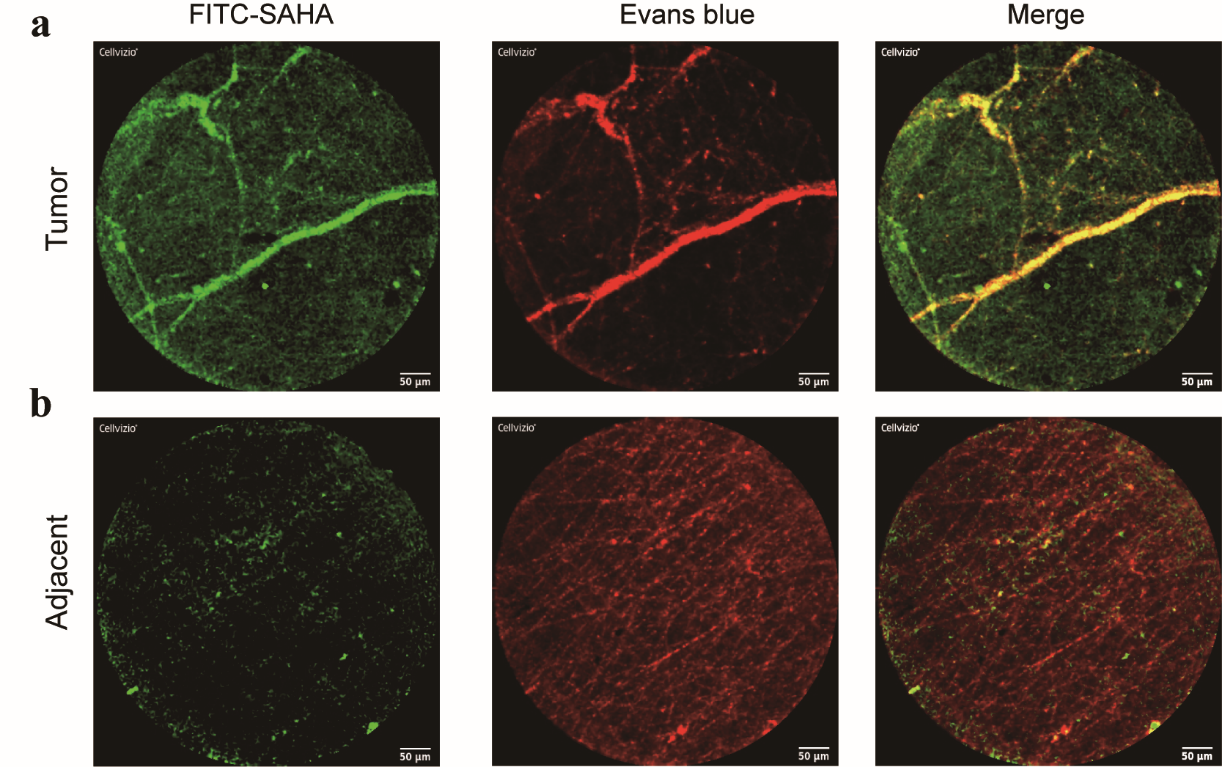


SFig.2 CLE imaging of GC tumor tissue and adjacent normal tissue. a The SAHA-FITC probe was enriched in gastric tumor sites. b In contrast, the para-tumor tissue shows only a weak background signal. The blood vessel was stained by Evans blue. A thick blood vessel and small blood vessels can be seen in the tumor and adjacent tissue respectively.

**SFig.3**


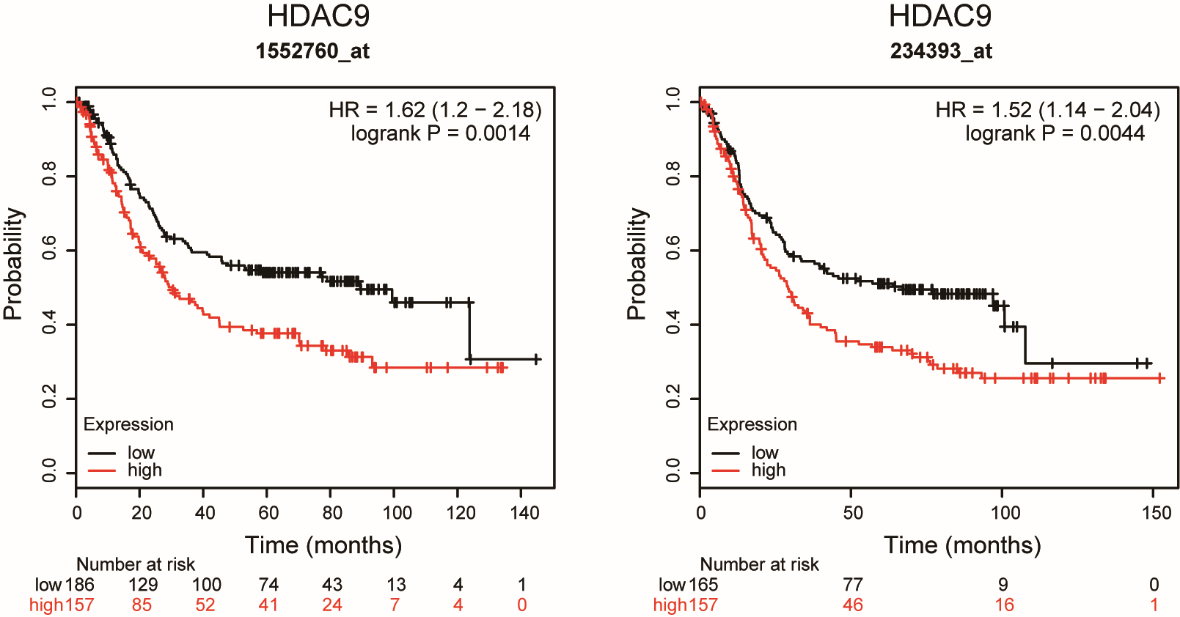


SFig.3 The prognostic effect of the expression of HDAC9 in www.kmplot.com. HDAC9 status was computed by using probe sets (1552760_at, 234393_at). All results showed that higher HDAC9 expression was correlated to worse prognosis.

**SFig.4**


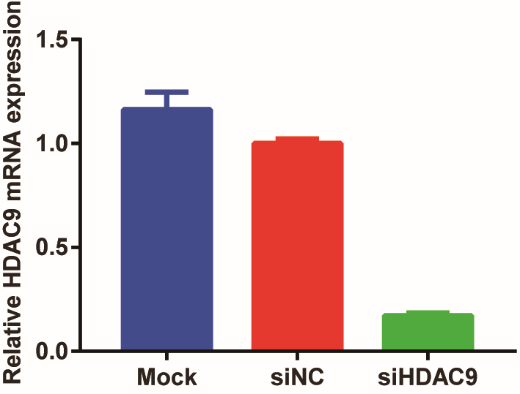


SFig.4 mRNA levels of HDAC9 after siHDAC9 transfection in BGC-823 cells was assessed by qRT-PCR.

**SFig.5**


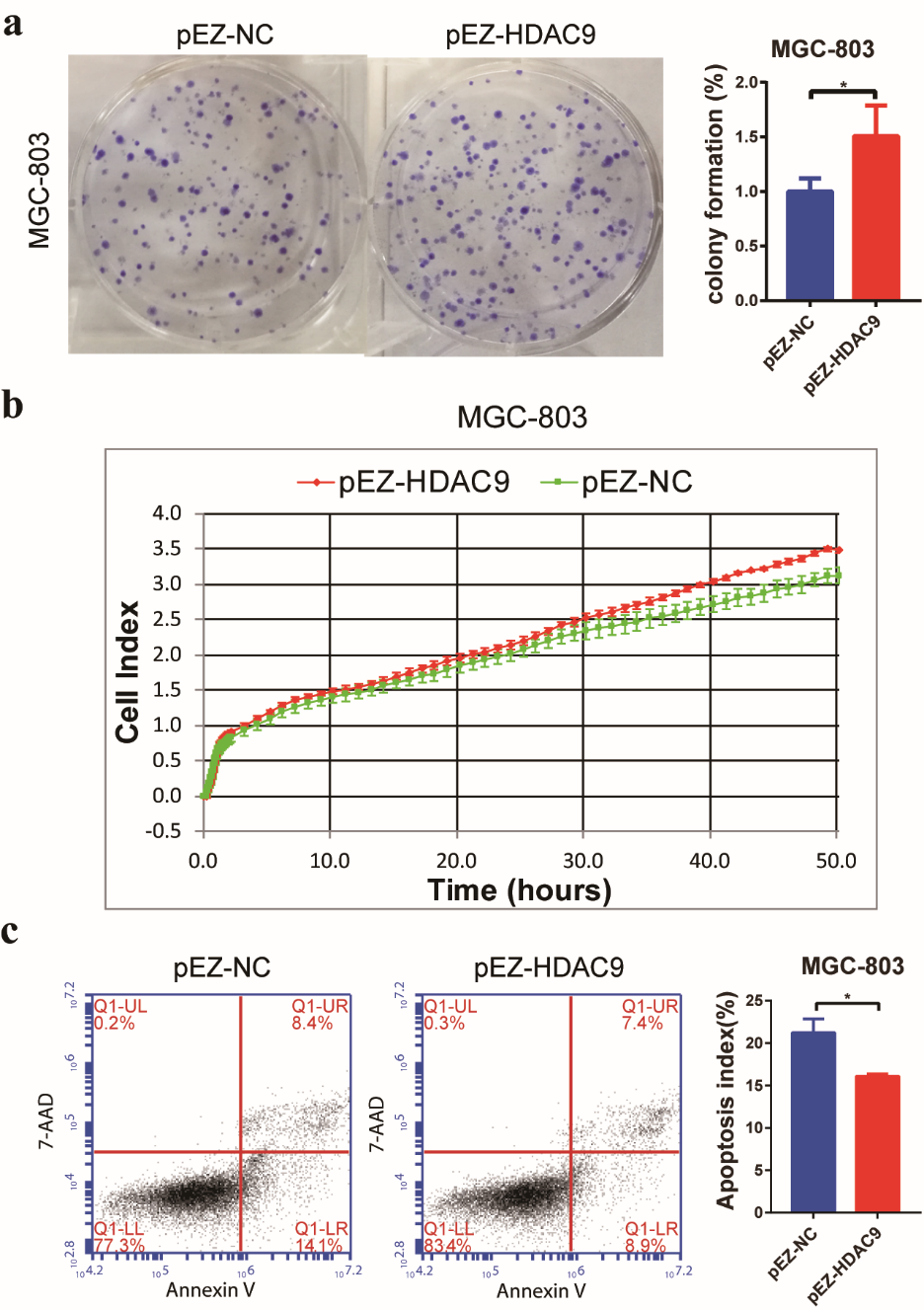


SFig.5 HDAC9 overexpression increased cell colony formation and proliferation and inhibited cell apoptosis. a MGC-803 cells transiently transfected with pEZ-HDAC9 showed higher colony formation capacity than cells transfected with negative vector (pEZ-NC). b Results showed a significantly higher rate of GC cell proliferation following transfection with pEZ-HDAC9 compared with cells transfected with pEZ-NC. c HDAC9 overexpression reduced apoptosis rate in GC cells by the Annexin V-PE/7-AAD assay. Error bars represent mean ± SD; * P<0.05, ** p<0.01 versus control group.

**SFig.6**


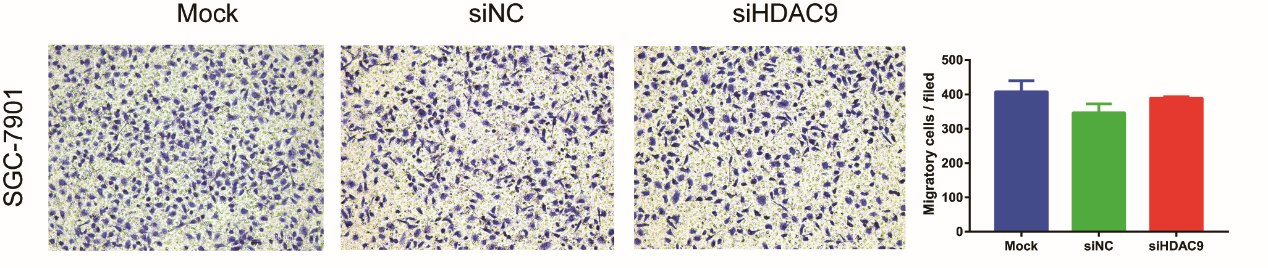


SFig.6 Representative images of transwell migration assay revealed HDAC9 silencing didn’t affect gastric cancer cell migration.
